# Letter from the Editor in Chief

**DOI:** 10.19102/icrm.2021.121107

**Published:** 2021-11-15

**Authors:** Moussa Mansour


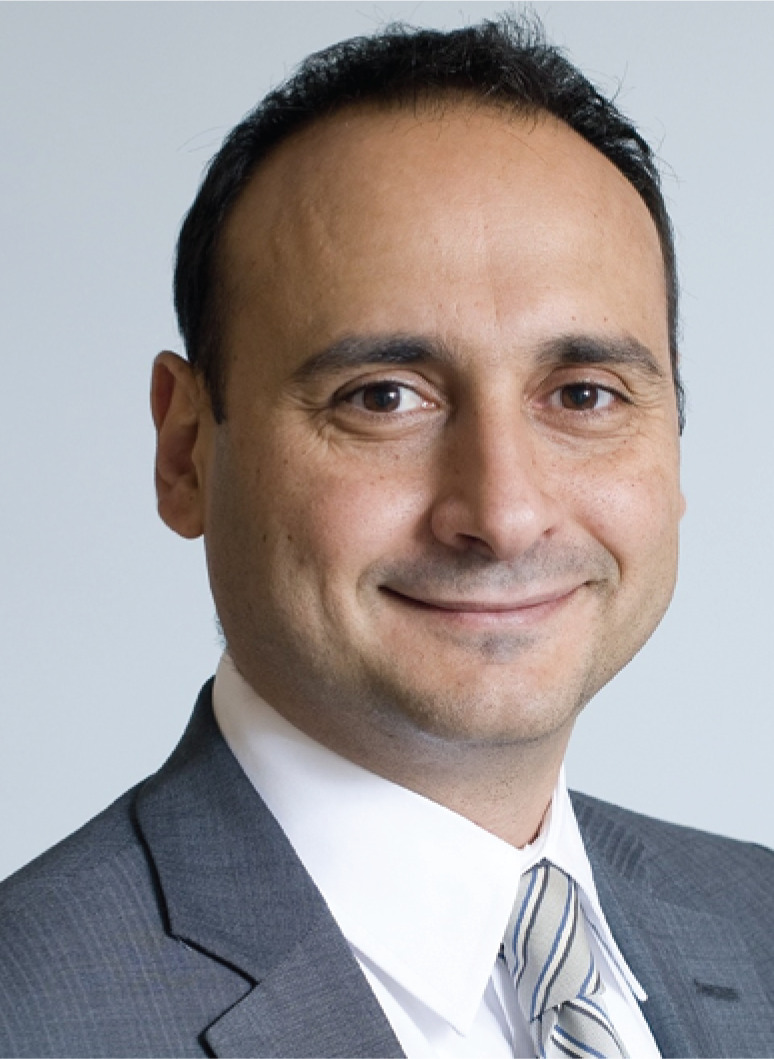


Dear Readers,

Esophageal injury is one of the most feared complications of ablation for atrial fibrillation (AF) and can occur with all forms of thermal energy. Over the years, many techniques and technologies have been introduced with the aim of lowering rates of esophageal injury, including luminal temperature monitoring, thermal imaging, esophageal deviation, and esophageal cooling, and there is a consensus that their use has been associated with a reduction in the frequency of this complication. However, one aspect of esophageal-protection devices that has not been well studied is their impact on the use of fluoroscopy imaging. Some tools, such as esophageal-deviation tools, require the use of X-ray imaging. Others, such as esophageal cooling, do not require imaging once the tool has been introduced in the esophagus.

This issue of *The Journal of Innovations in Cardiac Rhythm Management* contains an article titled “Impact of Active Esophageal Cooling on Fluoroscopy Usage During Left Atrial Ablation.”^1^ In this retrospective study, Zagrodzky et al. describe their experience with the use of esophageal cooling and report a 35% reduction in fluoroscopy time compared to that achieved using temperature monitoring. While their study has some limitations, including its retrospective design, it correlates with an important trend witnessed in the past few years, which is the desire to reduce the use of ionizing radiation during cardiac procedures.

Low- or zero-fluoroscopy ablation has been increasingly adopted into practice in many centers. Advanced electroanatomical mapping systems and intracardiac echocardiography have made this task achievable. As is the case in the abovementioned article, the ablation procedure will continue evolving to include measures to reduce fluoroscopy use. Moreover, I believe that fluoroscopy usage will be incorporated as a secondary outcome endpoint in future AF ablation studies.

I hope that you enjoy this issue of *The Journal of Innovations in Cardiac Rhythm Management*. On a separate note, I wish to welcome Dr. Melissa R. Robinson, clinical associate professor of medicine, medical director of the electrophysiology laboratory, and director of the complex ablation program at the University of Washington School of Medicine, who will serve going forward as the section editor of the journal’s “Innovations in VT” section.

Best wishes for a happy holiday season.

Sincerely,


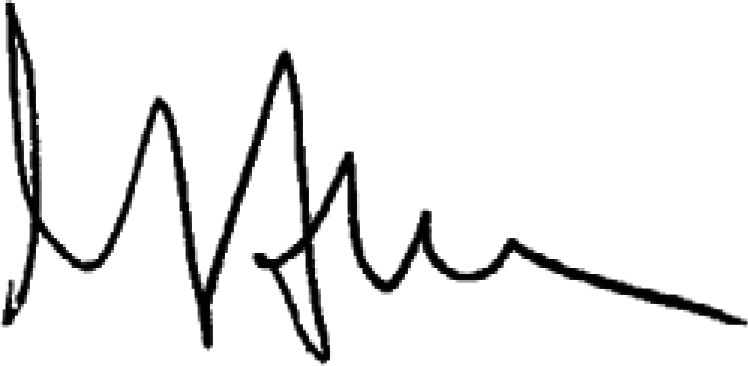


Moussa Mansour, md, fhrs, facc

Editor in Chief

*The Journal of Innovations in Cardiac Rhythm Management*

MMansour@InnovationsInCRM.com

Director, Atrial Fibrillation Program

Jeremy Ruskin and Dan Starks Endowed Chair in Cardiology

Massachusetts General Hospital

Boston, MA 02114
